# Cortical afferents onto the *nucleus Reticularis thalami* promote plasticity of low-threshold excitability through GluN2C-NMDARs

**DOI:** 10.1038/s41598-017-12552-8

**Published:** 2017-09-25

**Authors:** Laura M. J. Fernandez, Chiara Pellegrini, Gil Vantomme, Elidie Béard, Anita Lüthi, Simone Astori

**Affiliations:** 1 Department of Fundamental Neurosciences, University of Lausanne, 1005 Lausanne, Switzerland; 2 Present Address: Brain Mind Institute, École Polytechnique Fédérale de Lausanne, 1015 Lausanne, Switzerland

## Abstract

Thalamus and cortex represent a highly integrated processing unit that elaborates sensory representations. Interposed between cortex and thalamus, the *nucleus Reticularis thalami* (nRt) receives strong cortical glutamatergic input and mediates top-down inhibitory feedback to thalamus. Despite growing appreciation that the nRt is integral for thalamocortical functions from sleep to attentional wakefulness, we still face considerable gaps in the synaptic bases for cortico-nRt communication and plastic regulation. Here, we examined modulation of nRt excitability by cortical synaptic drive in Ntsr1-Cre x ChR2^tg/+^ mice expressing Channelrhodopsin2 in layer 6 corticothalamic cells. We found that cortico-nRt synapses express a major portion of NMDA receptors containing the GluN2C subunit (GluN2C-NMDARs). Upon repetitive photoactivation (10 Hz trains), GluN2C-NMDARs induced a long-term increase in nRt excitability involving a potentiated recruitment of T-type Ca^2+^ channels. In anaesthetized mice, analogous stimulation of cortical afferents onto nRt produced long-lasting changes in cortical local field potentials (LFPs), with delta oscillations being augmented at the expense of slow oscillations. This shift in LFP spectral composition was sensitive to NMDAR blockade in the nRt. Our data reveal a novel mechanism involving plastic modification of synaptically recruited T-type Ca^2+^ channels and nRt bursting and indicate a critical role for GluN2C-NMDARs in thalamocortical rhythmogenesis.

## Introduction

Much advancement has recently been achieved in the conceptual understanding of the *nucleus Reticularis thalami* (nRt). Strategically positioned along the thalamocortical path, the nRt is the major source of inhibition for thalamocortical cells of the dorsal thalamus^1^. Long described as a uniform sleep rhythm pacemaker, the nRt turns out to be involved not only in rhythmogenesis related to sleep, but also in wakefulness, memory and attention, and in orchestrating both local and brain-wide oscillatory patterns^2,3^. Moreover, previously described modulatory actions of nRt on sensory receptive fields^4^ turn out to be just one aspect of a broad and decisive role of nRt in guiding sensory attention^3,5,6^.

Afferents from cortical layers 5 and 6 are a source of strong glutamatergic excitation that influences the feedforward routing of thalamocortical activity during all arousal states. During wakefulness, corticothalamic layer 6 neurons augment their discharge to around 5–25 Hz during sensory stimulation^7–10^, and their activity sharpens the spatiotemporal properties of sensory receptive fields^11^ and promotes attentionally mediated sensory selection^5^. During non-REM (NREM) sleep or anaesthesia-induced thalamocortical rhythms, large sets of principal cortical cells including deep layers are synchronously activated^12^ and generate a phase-locking between cortical and thalamic rhythmic discharges^12–14^.

Sensory sectors of the nRt receive a prominent glutamatergic innervation from a subgroup of layer 6 pyramidal cells from the corresponding cortical sensory areas, which outnumbers inputs from thalamocortical (TC) neurons^15,16^. Cortico-nRt (corticoreticular) excitation typically overwhelms the direct corticothalamic excitation received by TC neurons, producing a marked feedforward inhibition in thalamus^13,17^. Short-term plasticity of the corticoreticular communication modifies the polarity and effectiveness of cortical control over thalamus, as demonstrated *in vitro* ^17^ and *in vivo* ^18^, which affects sensory adaptation^18^. Genetically induced long-term weakening in corticoreticular transmission leads to epilepsy^19^, whereas its strengthening affects sensory attention^5^. The basic elements underlying the strength of the corticoreticular feedforward projections comprise rapid rise times of excitatory potentials, marked short-term presynaptic facilitation^17,20^, high densities of postsynaptic AMPA receptors^16,21^, and a strong coupling of postsynaptic potentials to burst discharge^22,23^ via low-threshold T-type Ca^2+^ channels, which leads to rapid phasic inhibition of TC cells^2,24^. Ca_V_3.3 and Ca_V_3.2 channels are the T-type isoforms expressed in reticular cells: Ca_V_3.3 channels are mainly recruited by somatic depolarizations or single corticoreticular postsynaptic potentials, and are the major determinant of sleep spindle power^25^, whereas the role of Ca_V_3.2 in corticoreticular transmission and nRt rhythmogenesis remains to be clarified^23^.

A less explored element in corticoreticular communication is the NMDAR^20,26,27^, which appears to contribute only marginally to basal excitatory transmission^20,22^. Yet, synchronous and repetitive activation of cortical inputs may induce sufficient NMDAR activation and trigger plastic regulations of the corticoreticular feedback that outlast the already described short-term dynamics. Such activity-dependent long-lasting modulation is likely to be central for keeping up with behavioral demands of arousal states. Corticoreticular plasticity could also modify the operation of corticothalamic communication in the course of sleep, during which the dominance of thalamically generated rhythms, such as spindle and delta waves, varies along with the homeostatic and circadian regulation^28,29^. Moreover, learning during the day modifies sleep locally, such that thalamic rhythms can be strengthened^30–32^, which in turn depends on the efficiency of corticoreticular communication.

Here, we set out to define mechanisms of corticoreticular plasticity using optogenetic stimulation of cortical afferents onto sensory sectors of nRt in Ntsr1-Cre x ChR2^tg/+^ mice, that express the light-gated ion channel Channelrhodopsin2 (ChR2) selectively in corticothalamic layer 6 cells^33,34^. We found that photoactivated corticoreticular NMDAR currents are largely mediated by GluN2C-containing receptors (GluN2C-NMDARs), a subtype reported to be expressed in nRt cells and associated with pathophysiological thalamic rhythmogenesis^35–38^. In the slice preparation, repetitive photoactivation of corticoreticular synapses with brief 10 Hz trains induced an increase in nRt responsiveness to cortical inputs. Interestingly, the increased excitability resulted from the augmented recruitment of T-type Ca^2+^ channels, likely of the Ca_V_3.2 isoform, and was prevented by GluN2C-NMDAR blockade. In anaesthetized mice, stimulation of cortical afferents in the nRt with 10 Hz trains produced a frequency shift of cortical local field potentials (LFPs), with slow oscillation magnitude (SO, 0.5–1.25 Hz) being diminished and delta waves (δ, 1.25–4 Hz) becoming more dominant. The modulation of cortical rhythms was long-lasting and sensitive to local NMDAR blockade in the nRt.

Our results provide first evidence for a synaptic mechanism underlying nRt-dependent modulation of thalamocortical dynamics and highlight a critical role for GluN2C-NMDARs in setting the recruitment of low-threshold Ca^2+^ channels in thalamic rhythmogenesis.

## Results

### GluN2C-mediated transmission at corticoreticular synapses

**Figure 1:**
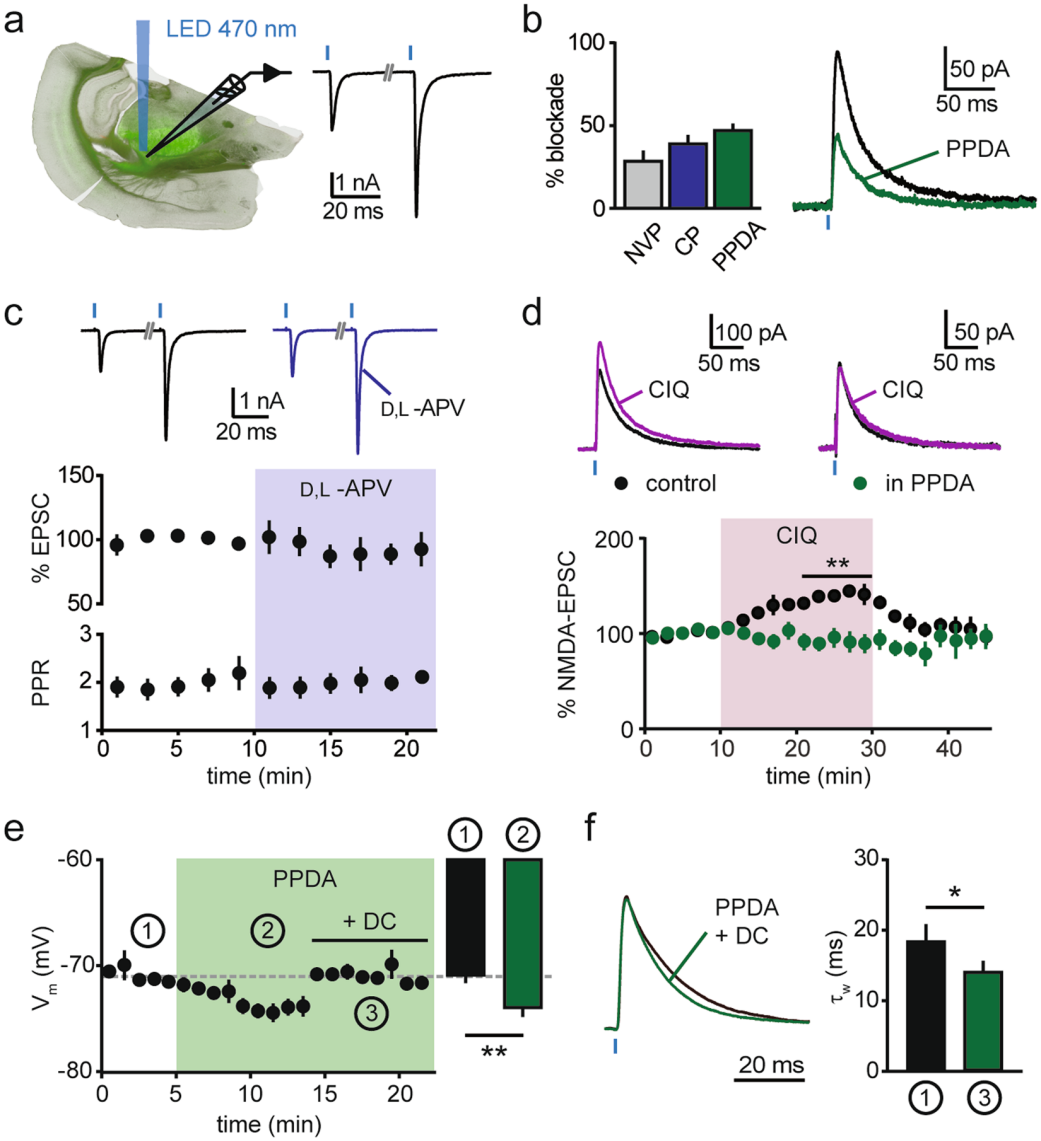
GluN2C-NMDARs at corticoreticular synapses. (**a**) Scheme of the experimental condition for optogenetic stimulation of corticoreticular synapses in acute slices of Ntsr1-Cre^tg/+^ mice. Green fluorescence in the image shows ChR2-positive fibers originating from layer 6 cortical cells and invading the thalamus. On the right, example EPSCs recorded at −70 mV showing pronounced paired-pulse facilitation (50 ms inter-stimulus interval). Blue thick marks indicate LED flashes. (**b**) Reduction of isolated corticoreticular NMDA-EPSCs upon superfusion of the GluN2A-preferring antagonist NVP-AAM077 (NVP, 50 nM, n = 7), the GluN2B-selective blocker CP-101.606 (CP, 10 µM, n = 7) and the GluN2C/2D blocker PPDA (500 nM, n = 7). On the right, example of mean NMDA-EPSCs recorded at +40 mV before (black) and after (green) PPDA superfusion. (**c**) Lack of effect of NMDAR blockade (100 µM D,L-APV) on peak amplitude and paired-pulse ratio (PPR) of AMPA-EPSCs recorded at −70 mV (n = 6), with example average traces at the top, before (black) and after (violet) D,L-APV perfusion. (**d**) The potentiating effect of the GluN2C/2D modulator CIQ (20 µM) on corticoreticular NMDA-EPSCs (n = 6) was prevented by PPDA (n = 6). Color-coded example traces are shown at the top. (**e**) Time course of the membrane potential of nRt cells recorded in current-clamp mode before (1) and after (2) superfusion of PPDA (n = 9). Membrane potential was reset to baseline values (dashed line) by direct current (DC) injection (3) for comparison of decay kinetics of EPSPs (in **f**). Bar graph represents average values in the indicated periods. (**f**) Left, mean EPSPs (normalized to peak) recorded before (black) and after (green) superfusion of PPDA. The membrane potential in PPDA was reset to baseline value by DC injection (time point 3 in panel **e**). Right, mean values of weighted τ (τ_w_) of EPSPs from double-exponential fit (n = 7). *p < 0.05, **p < 0.01, two-sided paired t test.

Neurons in the *nucleus Reticularis thalami* (nRt) express a complex subunit composition of NMDARs. At thalamoreticular synapses, GluN2B-NMDARs dominate over GluN2A-NMDARs throughout development, with an additional expression of the GluN2C subunit^35^. Here, we examined AMPAR- and NMDAR-mediated transmission at corticoreticular synapses by selectively photoactivating layer 6 afferents in acute slices from Ntsr1-Cre x ChR2^tg/+^ (Ntsr1-Cre^tg/+^) mice (Fig. 1). In nRt cells from 3–4 week-old animals, brief LED flashes (0.1–1 ms) elicited excitatory postsynaptic currents (EPSCs) characterized by a prominent short-term facilitation (paired-pulse ratio, PPR: 2.5 ± 0.2, n = 21; 50 ms inter-stimulus interval)(Fig. 1a), typical for corticothalamic inputs^22,35^. Compared to reported values for the NMDA/AMPA ratio at thalamoreticular synapses (~0.27)^35^, NMDA/AMPA ratio at cortical inputs was lower (0.13 ± 0.01, n = 21), consistent with the previously described minor contribution of NMDARs during basal corticoreticular transmission^20^. Isolated NMDA-EPSCs recorded at +40 mV were reduced by the GluN2A-preferring antagonist NVP-AAM077 (50 nM) and the GluN2B-selective blocker CP-101.606 (10 µM) (NMDA-EPSC decrease: 29.2 ± 5.8%, n = 7, p < 0.01; and 39.1 ± 5.3%, n = 7, p < 0.01, respectively, two-sided paired t test). The most pronounced blockade was induced by a low concentration of the GluN2C/GluN2D-preferring antagonist (2S*,3R*)-1-(Phenanthren-2-carbonyl)piperazine-2,3-dicarboxylic acid (PPDA, 500 nM, 47.0 ± 4.3%, n = 7, p < 0.01, two-sided paired t test; Fig. 1b). The high sensitivity to this compound was preserved in 6 week-old animals (51.8 ± 3.1%, n = 5, p < 0.05, two-sided paired t test). As PPDA may also inhibit GluN2A/2B-NMDARs^39^, although to a minor extent at 500 nM^40^, we further tested the recently developed GluN2C/2D inhibitor DQP-1105, which displays improved selectivity for recombinant GluN2C/2D subunits over GluN2A/2B subunits^41^. However, when applied on native NMDARs at corticoreticular synapses, this compound exhibited opposite effects depending on the dose used (Fig. S1): at 10 µM, DQP-1105 slightly inhibited NMDA-EPSCs (16.5 ± 10.5% reduction, n = 6, p = 0.06, two-sided paired t test), whereas, at 30 µM, DQP-1105 surprisingly potentiated NMDA-EPSCs in 4 out of 6 recordings (on average 44.1 ± 18.5% increase, n = 6, p > 0.05, two-sided paired t test). We ruled out non-specific effects of NMDAR blockade on glutamate release at cortical inputs, which might act as a confounding factor when measuring postsynaptic NMDA-EPSC inhibition. Bath application of the NMDAR-antagonist D,L-APV (100 µM) did not affect either the peak amplitude or the PPR of AMPAR-mediated EPSCs recorded at −70 mV (89.7 ± 8.7% of baseline amplitude; 101.9 ± 5.0% of baseline PPR, n = 6; p > 0.05 in both cases, two-sided paired t test; Fig. 1c), suggesting no involvement of presynaptic NMDARs in corticoreticular transmission under our recording conditions. To further demonstrate a specificity for the effects of PPDA, we tested the effect of the GluN2C/2D allosteric potentiator CIQ^42^. Corticoreticular NMDA-EPSCs were reversibly increased by CIQ application (20 µM, 39.6 ± 5.0%, n = 6, p < 0.01, two-sided paired t test). Strikingly, the CIQ-induced potentiation was abolished in PPDA-treated slices (−8.7 ± 9.2%, n = 6, p = 0.3, two-sided paired t test; Fig. 1d), indicating that CIQ exerts its action on a PPDA-sensitive population of NMDARs that is close-to-maximally blocked at 500 nM. Pharmacological tools to differentiate between native GluN2C- and GluN2D-NMDARs are currently not available. However, PPDA application did not change the decay kinetics of NMDA-EPSCs (τ_w_: 38.8 ± 6.4 ms vs. 37.0 ± 5.8 ms, n = 7, p = 0.22, two-sided paired t test). This excludes the involvement of slowly decaying GluN2D-NMDAR-mediated currents^43^, and is consistent with previous indications on the expression of synaptic GluN2C subunits in nRt cells obtained with pharmacological and genetic means^35,37^. The GluN2C-NMDARs dissected pharmacologically here likely underlie the previously found NMDAR-mediated component of the corticoreticular excitatory postsynaptic potentials (EPSPs) around resting membrane potentials^20^. Indeed, perfusion of PPDA in current-clamp recordings hyperpolarized nRt cells (from −71.0 ± 0.4 mV to −74.1 ± 0.7 mV, n = 9, p < 0.01, two-sided paired t test; Fig. 1e), confirming that the weakly Mg^2+^-sensitive GluN2C-NMDARs, likely extrasynaptic ones, are active at −70 mV^38^. Moreover, PPDA accelerated the decay times of corticoreticular EPSPs (τ_w_: 18.5 ± 2.3 ms vs. 14.2 ± 1.4 ms, n = 7, p < 0.05, two-sided paired t test; Fig. 1f), indicating that GluN2C-NMDARs can be recruited at synaptic sites by single stimulation at resting membrane potential.

### NMDAR-dependent plasticity at corticoreticular synapses

**Figure 2:**
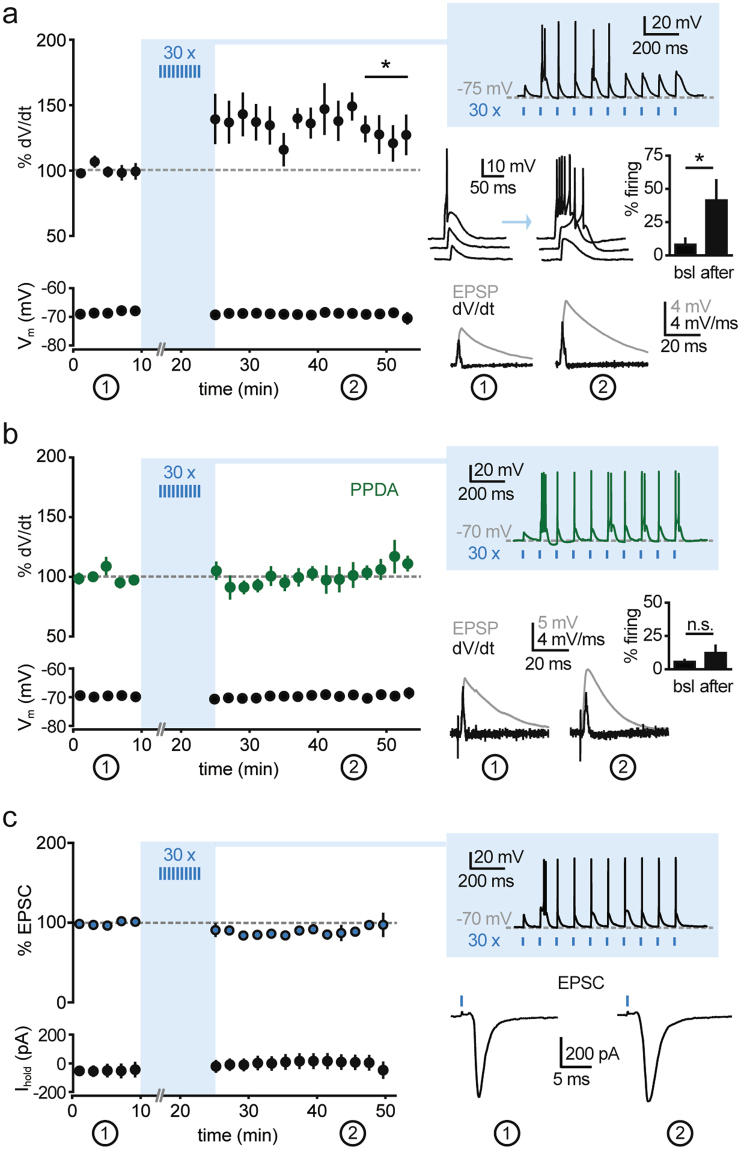
Repetitive corticoreticular activation induces a GluN2C-NMDAR-dependent increase in nRt excitability. (**a**) Left, time course of corticoreticular EPSP slope (analysed for % peak amplitude of the differential of the voltage signal, dV/dt) and of the membrane potential (V_m_) during baseline and after repetitive photostimulation (n = 6). Right: top, example response elicited by the train stimulation (10 Hz), which was repeated 30 times every 30 s; middle, examples of EPSPs during baseline and after induction, showing the increase in low-threshold firing, which is quantified in the bar graph (n = 9); bottom, example of average EPSPs and corresponding differential traces during baseline (1) and at the end of the recording (2). (**b**) Same representation as in (**a**) for experiments conducted in the presence of PPDA (500 nM, n = 8). (**c**) Left, time course of corticoreticular EPSCs recorded at −70 mV and of holding current (I_hold_) during baseline and after repetitive photostimulation (n = 6), indicating no change in AMPAR-mediated transmission. Right, examples of responses elicited during the train stimulation (performed in current-clamp) and average EPSCs recorded during baseline (1) and at the end of the recording (2). *p < 0.05, **p < 0.01, two-sided paired t test.

To explore the impact of more sustained corticoreticular activity at higher frequencies within the plausible *in vivo* range, we next asked whether repetitive activation (10 stimuli at 10 Hz) of cortical inputs could trigger proplastic functions of NMDARs at corticoreticular synapses. After recording baseline EPSPs around resting membrane potential (−60 mV/−70 mV) in the current-clamp mode, we elicited repetitive EPSPs while the postsynaptic neurons were hyperpolarized below −70 mV with somatic DC injections. This condition was chosen to reproduce membrane voltages typical for NREM sleep, thus favoring deinactivation of T-type Ca^2+^ channels and generation of low-threshold bursting upon stimulation of cortical afferents, typically appearing as a burst discharge on top of a triangular-shaped potential. Of note, the membrane potential measured at the soma might not reflect the actual voltage at dendritic sites. However, low-threshold Ca^2+^ currents are amplified at distal dendrites of nRt neurons, and bursts can be generated independently from the soma^22^. Repetitions of 10 Hz trains (30 times every 30 s) increased postsynaptic excitability: whereas single stimulation during baseline induced subthreshold EPSPs that were only occasionally crowned by spikes (7.9 ± 5.5%, n = 9) at −60 mV, after train stimulation the same photoactivation provoked firing at a higher rate (41.3 ± 16.0%, p < 0.05, two-sided paired t test; Fig. 2a). We quantified changes in EPSP waveform in a subgroup of recordings in which the elicited action potentials occurred after EPSP peak and did not contaminate the initial slope of the response. EPSP slopes were quantified through measuring the maximal slope (peak of the voltage derivative), which increased by 31.9 ± 11.3% in the time window 20–30 min after train stimulation as compared to baseline condition (n = 6, p < 0.05, two-sided paired t test; Fig. 2a).

To probe the role of GluN2C-NMDARs in this form of plasticity, the train-induced potentiation was repeated in the presence of PPDA. Under these conditions, the potentiation of spike propensity was prevented (5.1 ± 8.8% increase in the derivative of the voltage responses and no change in firing rate, n = 8, p > 0.05, two-sided paired t test; Fig. 2b), indicating that GluN2C-NMDARs contribute to induce plasticity at corticoreticular synapses.

**Figure 3:**
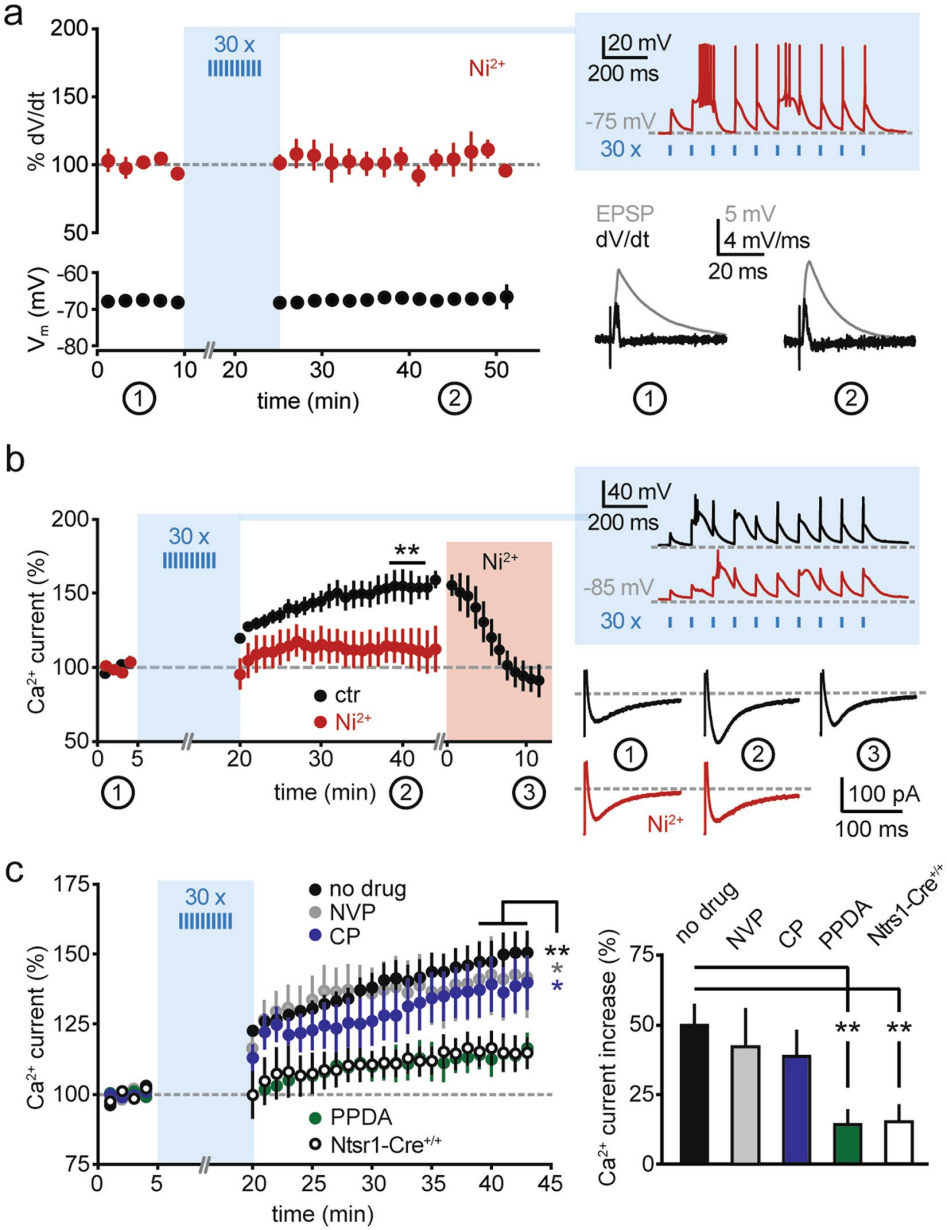
GluN2C-NMDAR-dependent increase in low-threshold Ca^2+^ currents. (**a**) Left, time course of corticoreticular EPSP slope (analysed for % peak amplitude of the differential of the voltage signal, dV/dt) and of the membrane potential (V_m_) during baseline and after repetitive photostimulation in the presence of Ni^2+^ (100 µM, n = 6). Right, example response elicited by the train stimulation and average EPSPs with corresponding differential traces during baseline (1) and at the end of the recording (2). Of note, nRt bursting during the induction protocol was not blocked by 100 µM Ni^2+^. However, Ni^2+^ prevented EPSP potentiation. (**b**) Left, time course of low-threshold Ca^2+^ currents elicited at the offset of brief hyperpolarizations (from −50 mV to −100 mV, 125 ms) during baseline and after repetitive corticoreticular stimulation. The series ‘ctr’ (black, n = 6) contains a subset of recordings from panel (**c**) (4 cells from the group ‘no drug’, 2 cells from the group ‘NVP’), in which 100 µM Ni^2+^ was superfused at the end of the recording. The series ‘Ni^2+^’ (red, n = 7) contains recordings performed in the constant presence of 100 µM Ni^2+^. Right, example responses elicited by the train stimulation and average Ca^2+^ currents in the time windows indicated by the numbers. (**c**) Left, time course of Ca^2+^ currents during baseline and after repetitive corticoreticular stimulation in control experiments (no drug, n = 9), in the presence of NVP-AAM077 (NVP, 50 nM, n = 6), in the presence of CP-101.606 (CP, 10 µM, n = 7), in the presence of PPDA (500 nM, n = 6), and in ChR2-negative mice (Ntsr1-Cre^+/+^, n = 6). Right, average increase in Ca^2+^ currents in the different recording conditions. *p < 0.05, **p < 0.01, two-sided paired t test; **p < 0.01, Mann-Whitney test for panel (**c**).

As a possible mechanism of expression of this form of plasticity, we first considered an increase in the contribution of postsynaptic AMPARs. Surprisingly, postsynaptic currents recorded at −70 mV in voltage-clamp mode, mainly reflecting AMPAR-mediated transmission, were not affected by the repetitive 10 Hz stimulation, which was provided, as before, in current-clamp mode (5.9 ± 6.1% change in amplitude vs. baseline, n = 6, p > 0.05, two-sided paired t test; Fig. 2c). As corticoreticular stimulation often generates low-threshold spiking via Ca_V_3 Ca^2+^ channels (Fig. 2a), we then asked whether the train stimulation could augment the recruitment of Ca_V_3 channels to the postsynaptic potentials. To test this, we reduced the contribution of low-threshold Ca^2+^ channels, while preserving burst discharges of nRt cells during the induction protocol, by using a low concentration of Ni^2+^ (100 µM), which preferentially blocks Ca_V_3.2 channels^2,44^. In this condition, although Ca_V_3.3-mediating bursting during the train stimulation was still generated (Fig. 3a), no potentiation of postsynaptic responses occurred (3.7 ± 10.3% increase in the derivative of the voltage responses, n = 6, p > 0.05, two-sided paired t test).

To directly test whether repetitive photoactivation increases Ca_V_3 channel contribution, we elicited low-threshold Ca^2+^ currents by brief somatic hyperpolarizing steps applied to nRt cells voltage-clamped at −50 mV (Fig. 3b,c). To prevent non-clampable action currents during Ca^2+^ current activation, we included the Na^+^ channel blocker QX-314 in the intracellular solution. Train stimulation of cortical inputs in current-clamp mode (in cells hyperpolarized below −70 mV) increased the peak amplitude of whole-cell Ca^2+^ currents (by 49.7 ± 7.6%, n = 9, p < 0.01, two-sided paired t test; Fig. 3c). This potentiation was sensitive to Ca_V_3.2 channel blockade. First, residual Ca_V_3.3-mediated currents elicited in the presence of Ni^2+^ were not modified by train stimulation (14.0 ± 11.7%, n = 7, p > 0.05, two-sided paired t test; Fig. 3b). Second, established levels of potentiation could be reversed by Ni^2+^ application at the end of the recording, which reset the peak amplitudes to baseline values (from 52.5 ± 9.8% to −4.8 ± 8.5%, n = 6, p < 0.01, two-sided paired t test; Fig. 3b). Next, we confirmed the involvement of GluN2C-NMDARs (Fig. 3c). The potentiation of low-threshold Ca^2+^ currents still occurred in the presence of the GluN2A-preferring blocker NVP-AAM077 (41.9 ± 14.1%, n = 6, p < 0.05, two-sided paired t test), and of the GluN2B-selective blocker CP-101.606 (38.5 ± 9.5%, n = 7, p < 0.05, two-sided paired t test), but was prevented by GluN2C receptor blockade with PPDA (14.2 ± 5.5%, n = 6, p > 0.05, two-sided paired t test). The extent of Ca^2+^ current increase in PPDA was comparable to the slight increase observed in Ntsr1-Cre^+/+^ mice lacking ChR2 expression (14.2 ± 5.5%, n = 6, p > 0.05, two-sided paired t test), indicating that Ca^2+^ currents may be affected by the hyperpolarization applied during the induction protocol or by the prolonged whole-cell recording. However, the % changes in these series were significantly different from the level of potentiation reached in Ntsr1-Cre^tg/+^ mice with no drug (Mann-Whitney test, p < 0.01; Fig. 3c).

Altogether, these data indicate that repetitive photoactivation of cortical afferents increases postsynaptic responses and excitability of nRt cells. This form of plasticity requires GluN2C-NMDARs for its induction and results in the augmented contribution of Ca_V_3 channels, most likely of the Ca_V_3.2 subtype, to postsynaptic excitability. Importantly, low-threshold Ca^2+^ currents isolated in voltage-clamp can be potentiated independently of postsynaptic potentials, indicating that the augmented recruitment of Ca_V_3 channels in current-clamp recordings is not a consequence of increased glutamatergic drive, but is due to a modification in Ca_V_3 channel expression or activation properties.

### Repetitive corticoreticular activation modulates cortical oscillations

**Figure 4:**
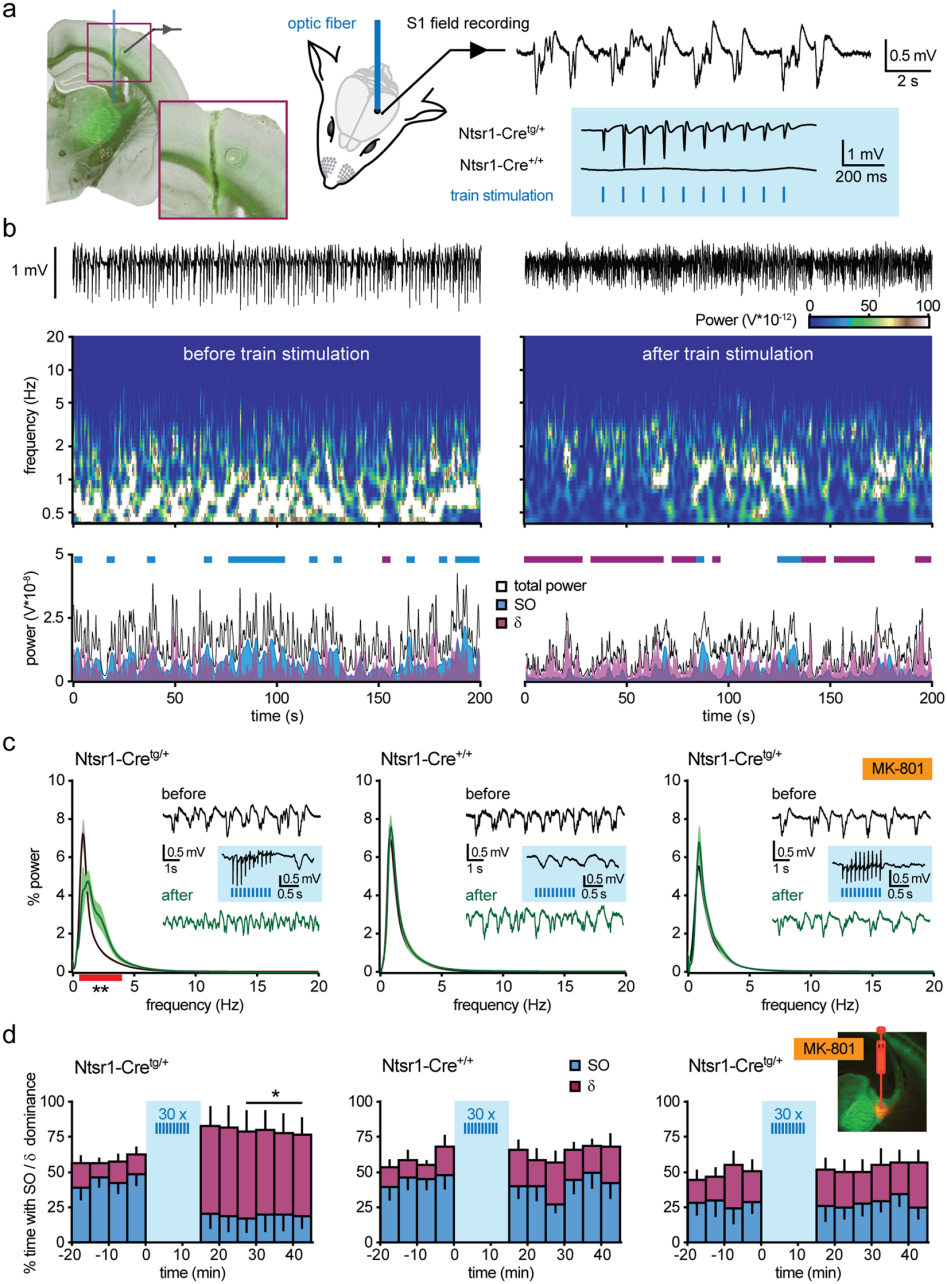
NMDAR-dependent modulation of thalamocortical oscillations *in vivo*. (**a**) Left, experimental settings for *in vivo* optogenetic stimulation of corticoreticular fibers. Inset shows the trajectory of the optic fiber positioned on top of the nRt, and the electrocoagulation marking the position of the electrode in S1. Right, example of cortical LFPs showing low-frequency oscillations typical for urethane anaesthesia, and representative responses to laser stimulation elicited in Ntsr1-Cre^tg/+^ mice, whereas Ntsr1-Cre^+/+^ mice lacked evoked responses. (**b**) Raw cortical LFP traces (top), power spectrum from wavelet analysis (middle) and power diagram for total power, for SOs (0.5–1.25 Hz) and δ oscillations (1.25–4 Hz) (bottom) during a 200-s long period, before and after train stimulation. Thick lines in the power diagram represent epochs in which SOs (blue) or δ oscillations (purple) were dominant, i.e. contribute to ≥ 50% of the total power. (**c**) Mean power spectra during baseline (black) and at 10–30 min after train stimulation (green) in Ntsr1-Cre^tg/+^ (n = 7, left), Ntsr1-Cre^+/+^ (n = 5, middle), and Ntsr1-Cre^tg/+^ mice with local injection of MK-801 (n = 6, right). Red thick line indicates the frequency range where a significant difference occurred (**p < 0.01, *post hoc* Fisher’s LSD test after two-way repeated measures ANOVA on 1-Hz-binned values). Representative cortical LFP stretches and photoactivated responses are displayed in the insets. (**d**) Time course of % dominance of SOs (blue) and δ oscillations (purple) in the three experimental series shown in (**c**). Fluorescence image shows verification of the injection site for MK-801 with Alexa 594. *p < 0.05, Wilcoxon test.

We asked whether repetitive photoactivation of corticoreticular afferents as performed *in vitro* could effectively modulate thalamocortical excitability in the intact animal. In Ntsr1-Cre^tg/+^ mice anaesthetized with urethane (2-2.5  mg/g i.p.) we monitored cortical oscillations by recording local field potentials (LFPs) in the somatosensory cortex S1 (Fig. 4a). Urethane anaesthesia was chosen as it allows for prolonged recordings and it generates both periods of highly synchronized brain oscillations and periods of lower synchronization and higher excitability, which has been suggested to reproduce the physiological sequence of NREM and REM sleep^45,46^. During baseline recordings performed >1.5 h after urethane injection, cortical LFPs displayed high occurrence of synchronized oscillations in the low frequency range (0.5–1.25 Hz; slow oscillations, SOs), which dominated the power spectrum for the majority of the recording time (i.e. SO power contributed by at least 50% to the total power calculated in 4-s epochs; Fig. 4b–d). We then stimulated ChR2-expressing corticothalamic fibers with brief laser flashes (0.5 ms) delivered with an optic fiber positioned above the nRt. The resulting activation of the thalamocortical system was demonstrated by the phasic responses appearing in S1 (Fig. 4a). Repetitions of 10 Hz train stimulations (30 times, as performed in the experiments *in vitro*) resulted in a long-lasting modification of the spectral profile of cortical LFPs. The pronounced peak in the 0.5–1.25 Hz frequency range in the LFP power spectrum was strongly decreased, while an additional component appeared in the delta frequency range (δ, 1.25–4 Hz) (two-way repeated measures ANOVA, F_17, 108_ = 8.59, p < 0.0001 for frequency x stimulation interaction, Fig. 4c). Consistently, the occurrence of episodes dominated by SOs substantially diminished (from 46.7 ± 7.9% during baseline to 18.5 ± 10.6% during the last 20 min of recording; n = 7, p = 0.08, Wilcoxon test), whereas periods dominated by δ oscillations occurred throughout the post-induction phase (13.0 ± 5.5% during baseline vs. 59.9 ± 13.6% during the last 20 min of recording; n = 7, p < 0.05, Wilcoxon test; Fig. 4d). Thalamocortical oscillations were not affected by the prolonged recording sessions, nor modified by the laser stimulation *per se*, as the power spectrum and the occurrence of SOs and δ oscillations were on average unaltered in Ntsr1-Cre^+/+^ mice not expressing ChR2 (n = 5, two-way repeated measures ANOVA, F_17, 72_ = 0.822, p > 0.05 for frequency x stimulation interaction; Fig. 4c,d).

To investigate the involvement of NMDARs at corticoreticular synapses, we tested the effect of local blockade with the irreversible open channel blocker MK-801, which was injected in the nRt prior to the positioning of the optic fiber (2 injection sites, 4 µg of MK-801 each). Correct targeting of the drug was verified by concomitant injection of Alexa 594 (Fig. 4d). With MK-801 injection, but not in control experiments with vehicle injection (Fig. S2a), the photoactivated modulation of cortical LFP power spectrum was prevented (two-way repeated measures ANOVA, F_17, 90_ = 1.13, p > 0.05 for frequency x stimulation interaction, Fig. 4c) and the relative dominance of SOs and δ oscillations was not affected (from 27.6 ± 9.7% and 21.7 ± 7.4% during baseline to 29.1 ± 7.5% and 25.9 ± 8.4% during the last 20 min of recording, for SOs and δ oscillations, respectively, n = 6, p > 0.05, Wilcoxon test; Fig. 4d). These results were replicated in experiments with systemic application of MK-801 (2 mg/kg), in which neither the power spectrum (two-way repeated measures ANOVA, F_17, 72_ = 0.79, p > 0.05 for frequency x stimulation interaction) nor the contribution of SOs and δ oscillations was changed (dominance: from 19.1 ± 6.4% and 25.6 ± 5.6% during baseline to 19.4 ± 8.0% and 35.9 ± 10.8% during the last 20 min of recording for SOs and δ oscillations, respectively, n = 5, p > 0.05, Wilcoxon test; Fig. S2b). Of note, NMDAR blockade appeared to affect thalamocortical oscillations under baseline condition, as the contribution of SOs to the total power spectrum with MK-801 was lower as compared to drug-free recordings (Fig. 4d and Fig. S2). However, blockade of thalamic NMDARs did not induce a shift in the baseline LFP power spectrum (Fig. 4c,d), suggesting that the effects of cortical photoactivation could not be occluded by MK-801.

Altogether, these data indicate that repetitive corticoreticular stimulation *in vivo* induces a long-term, NMDAR-dependent modulation of thalamocortical oscillations, consistent with the NMDAR-dependent alterations in nRt excitability described *in vitro*.

## Discussion

Our work identifies a novel form of synaptic plasticity between cortex and nRt that specifically requires GluN2C-NMDARs on nRt cells. This plasticity is expressed through a potentiated recruitment of T-type Ca^2+^ channels via corticoreticular synapses, resulting in enhanced firing of nRt cells in response to cortical glutamate release. Through analogous plasticity-promoting stimulation *in vivo*, we induced a long-term modification of thalamocortical synchronization in the corresponding areas of the anaesthetized somatosensory cortex that also depends on NMDAR activation in the nRt. Together, this work suggests that GluN2C-NMDARs can augment nRt responsiveness to cortical inputs in the long-term, which affects cortical synchronization related to states of low arousal, in our case anaesthesia.

The involvement of GluN2C-NMDARs is a unique aspect of this plasticity. The thalamus is one of the forebrain structures most strongly expressing this NMDAR subtype^47,48^. Compared to the GluN2A- and GluN2B-NMDARs that predominate at forebrain synapses, GluN2C-NMDARs were so far described most extensively in cerebellar granule cells, where they are recruited by mossy fiber inputs both synaptically and extrasynaptically and are implicated in cerebellar synaptic plasticity^49,50^. The role of GluN2C-NMDARs in non-cerebellar brain areas, including nRt, has been based on GluN2C-deficient mice or GluN2C-expressing cultured cells^37,51,52^, owing to the poor selectivity of GluN2-directed pharmacological tools^43,53^. Recently developed subtype-specific drugs provide new impetus in advancing the field^54,55^. In our case, the allosteric potentiator for GluN2C/2D subunits CIQ augmented NMDAR-EPSCs, and its effect was antagonized by PPDA at concentrations that predominantly block GluN2C/2D-NMDARs. Considering the slow decay of currents carried by GluN2D-NMDARs, which was not seen in our recordings, and the low thalamic expression of the GluN2D subunit after the first postnatal days^38,56^, our data indicate that GluN2C-NMDARs are recruited by corticoreticular synapses, as already suggested by comparison of current-voltage relationships between wild-type and GluN2C-knock out mice^37^. Even if GluN2A-NMDARs were partially blocked by PPDA, the extent of its effect - on isolated NMDAR-EPSCs and on Ca^2+^ current potentiation - compared to the GluN2A-preferring antagonist NVP-AAM077^57,58^ still argues for an unusually large synaptic GluN2C component. In our acute slice recordings, we did not observe consistent effects with DQP-1105, a compound that displays high selectivity for recombinant rat and human GluN2C/2D channels^41^. It has to be mentioned that NMDAR properties, including pharmacological sensitivity, can be modified by the presence of triheteromeric NMDARs^43,53^, and this is also the case for GluN2C subunits that can coassemble into triheteromers with GluN2A^49,59^. Therefore, the pharmacological profile we present strongly argues for native GluN2C-NMDARs at corticoreticular synapses, although it remains open whether GluN2C is integrated into tri- or diheteromers.

GluN2C-NMDARs display low sensitivity to Mg^2+^, which implies that they can open around resting membrane potentials^43^. We showed previously that PPDA, but not CP-101.606, decelerates the decay kinetics of EPSCs recorded at −70 mV at thalamoreticular synapses^35^, consistent with a GluN2C-mediated component. Here, we show that corticoreticular EPSPs also contain a PPDA-sensitive component when elicited from a comparable potential. In addition, we observed a hyperpolarizing action of PPDA that is consistent with a tonic NMDAR-current active at resting membrane potential, which was proposed to modulate nRt excitability^38^.

A further atypical aspect of the GluN2C-NMDAR-mediated plasticity is the mechanism of expression, as it does not involve a potentiation in postsynaptic glutamate receptors, but an increase in low-threshold Ca^2+^ currents. Moreover, the plasticity affects not only local Ca^2+^ spikes recruited by synaptic stimulation (Fig. 2a), but also extends to whole-cell currents elicited by somatic hyperpolarization (Fig. 3b,c), which is likely to globally enhance the responsiveness of nRt cells to glutamatergic inputs, including tonic forms, such as those mediated by the GluN2C-NMDARs shown here. The intracellular mechanisms leading to the enhanced contribution of Ca^2+^ currents remain to be elucidated. Potentiation of low-threshold Ca^2+^ currents was prevented by low concentrations of Ni^2+^, which preferentially blocks Ca_V_3.2 channels. Interestingly, the Ca_V_3.2 isoform exhibits features that favor use-dependent regulation, such as high sensitivity to redox agents and intracellular kinases^60^. In addition, there are indications for a bidirectional regulation of Ca_V_3.2 channels and NMDARs. On the one hand, Ca_V_3.2-mediated local Ca^2+^ influx can regulate synaptic NMDAR transmission in an activity-dependent manner^61^. On the other hand, Ca^2+^ influx initiated by NMDARs can activate intracellular cascades that phosphorylate Ca_V_3.2 channels and induce a shift in the activation curve that facilitates their recruitment^62^.

Altered burst discharge propensity in thalamus as a result of activity-dependent forms of synaptic plasticity has been reported for the reciprocal synapses between nRt and TC cells, and involves persistent changes in synaptic strength of inhibitory nRt-TC synapses^63,64^ and excitatory thalamoreticular synapses^35^. A presynaptically expressed form of plasticity at corticothalamic synapses that strengthens glutamate release has also been described^65^. However, in these reports, the consequences on rhythm generation in the *in vivo* situation were not addressed. In the current study we show that synaptic plasticity at the level of thalamus is highly relevant for the state of synchronization in cortex. The exact consequences of this plasticity on thalamus need to be further elaborated through direct recordings of nRt and TC cells. We speculate that the corticoreticular plasticity described here enhances nRt excitability and induces a net increase in the inhibitory drive onto TC neurons. The resulting hyperpolarization of TC cells drives intrinsic thalamic clock-like oscillations in the δ range - which is consistent with the shift in the spectral landscape observed in our recordings in anesthetized animals and which is reminiscent of the δ wave promotion through direct stimulation of the nRt^66^. Corticoreticular plasticity may co-occur with TC cell intrinsic plasticity triggered by low-frequency TC bursting (<1 Hz, shown by Sieber *et al*.^64^), also resulting in increased inhibitory drive, to promote the shift from cortical slow oscillations to δ rhythm during sleep. On the other hand, the plasticity described by Pigeat *et al*.^63^ might represent a subsequent mechanism to exit from δ oscillations, as it involves depression of GABAergic currents in TC cells upon synaptic activation in the δ range (1.6 Hz).

Previous optogenetic manipulations of nRt activity *in vivo* relied on direct stimulation of reticular neurons in vesicular GABA transporter (VGAT)-ChR2 mice, which could reproduce sleep spindles or rapidly increase slow-wave activity in the δ range, depending on the intensity and duration of the stimulus^66,67^. Here, we show that promotion of δ oscillations by the nRt can be effectively induced by a form of plasticity that is triggered synaptically rather than through direct somatic excitation. Phasic stimulation (<1 ms) of glutamatergic cortical afferents appears to be more effective than direct tonic photoactivation of nRt cells, as the resulting alterations in thalamocortical oscillations were not only transient^66,67^, but lasted for at least 30 min after induction. Thus, our results extend recent data showing that corticoreticular communication varies in the short-term and is important for thalamic excitability^18,22^. The effect we present here is likely to be very powerful, given that we found it to overcome a drug-induced thalamocortical synchrony and to promote a shift towards activity at higher frequencies. It also argues in favor of a broader role for thalamic plasticity as potential mechanism whereby the spectral composition of sleep can be modulated. During sleep and states of anaesthesia, a primary effect of layer 6 input is to recruit and synchronize thalamically generated rhythms into the sequence of thalamocortical waves typical for these low-arousal states^68^. For example, sleep spindles occur preferentially during activated states of the slow oscillation^12,68,69^ and can be triggered by burst discharge in nRt^45,67^, presumably through non-NMDA-mediated EPSPs at cortico-nRt synapses^20,70^. Furthermore, mGluR-dependent activation of nRt cells through cortical afferents can recruit thalamus into slow wave generation (for review, see^71^). Strengthening of corticoreticular inputs, thus, seems to be a key mechanism through which thalamic components of sleep and anaesthesia could be modified. In our conditions, the urethane-induced attenuation of thalamocortical activity might have favored the modulation of low-frequency components of the spectrum rather than spindle waves. Therefore, similar experiments conducted in sleeping animals are needed to determine whether sleep spindle generation can also be modified as a result of corticoreticular plasticity. Another interesting aspect concerns the topology of corticoreticular plasticity and of the resulting δ wave appearance across cortical areas. As corticothalamic cells located in the upper and lower layer 6 differentially innervate nRt regions and dorsal thalamic nuclei^15^, it is possible that localized photostimulation in specific portions of the layer 6 will have a different impact on both the intensity and the spatial extent of cortical wave modulation.

Our parallel *in vitro* and *in vivo* approaches indicate that GluN2C-NMDAR-mediated synaptic excitation is integral for a flexible corticoreticular communication. Therefore, we propose that the plasticity described here could be a potential key regulator for various forms of thalamocortical crosstalk during arousal states. We also propose that impaired corticoreticular plasticity due to deficient coupling between GluN2C-NMDARs and low-threshold Ca^2+^ channels might be a factor in pathophysiological conditions involving nRt excitability^5,72^. Notably, GluN2C expression is decreased at thalamic sites in schizophrenic patients^73,74^, who also exhibit specific alterations in nRt rhythmogenesis, i.e. a reduction in sleep spindles^75^. Our results bring further evidence to the link between thalamic NMDAR hypofunction and the emergence of aberrant thalamic oscillations related to schizophrenic symptoms^38^, and are also in line with the proposed role of low-threshold Ca_V_3 channel dysfunction in schizophrenia^76,77^.

## Methods

### Animal handling

All animal care procedures and experimental methods were in accordance with the Swiss Federal Guidelines for Animal Experimentation and were approved by the Veterinary Office of the Canton de Vaud. Animals were kept in the institute’s animal facility with food and water *ad libitum*. Female homozygous B6;129S-Gt(ROSA)26Sort^m32(CAG-COP4*H134R/EYFP)Hze/J^ mice (Ai32^34^), were crossed with tg/+ males from the Ntsr1-Cre line (B6.FVB(Cg)-Tg(Ntsr1-cre)GN220Gsat/Mmucd) line^33^, yielding Cre-positive animals expressing ChR2 in cortical layer 6 pyramidal cells (Ntsr1-Cre x ChR2^tg/+^) and Cre-negative control littermates (Ntsr1-neg x ChR2^tg/+^), referred to as Ntsr1-Cre^tg/+^ and Ntsr1-Cre^+/+^, respectively.

### *In vitro* electrophysiological recordings

Acute horizontal brain slices (300 μm-thick) were prepared from animals of either sex, as previously described^25^, between postnatal day P18 and P28, unless otherwise indicated (P40–48 for PPDA effect in older mice). In the recording chamber, slices were constantly superfused with oxygenated artificial CSF (ACSF) at 30–32 °C containing (in mM): 125 NaCl, 25 NaHCO_3_, 2.5 KCl, 1.25 NaH_2_PO_4_, 1.2 MgCl_2_, 2 CaCl_2_, 25 glucose, 1.7 L( + )-ascorbic acid, 0.01 glycine, 0.1 picrotoxin. Visually identified nRt neurons were whole-cell patched with borosilicate glass pipettes (TW150F-4, WPI). For voltage-clamp recordings with NMDAR pharmacology, pipettes (3–4 MΩ) were filled with (in mM): 127 CsGluconate, 10 HEPES, 2 BAPTA, 6 MgCl_2_, 2 Mg-ATP, 0.2 Na-GTP, 10 phosphocreatine, 2.5 QX-314 (290–300 mOsm, pH 7.2–7.3). A liquid junction potential of −8 mV was corrected for. After recording AMPAR-mediated responses at −70 mV, the membrane potential was slowly switched to +40 mV, and DNQX (0.01–0.04 mM) was added to the perfusate to isolate the NMDAR-mediated current. GluN2-directed blockers - NVP-AAM077 (Novartis Pharma), CP-101.606 (Pfizer Pharmaceuticals), PPDA and DQP-1105 (Tocris Bioscience) - were superfused after > 8 min of stable baseline, and their action was assessed after > 5 min of a steady blockade. Current-clamp and plasticity recordings were performed with pipettes (2–3 MΩ) filled with an intracellular solution containing (in mM): 140 KMeSO_4_, 10 KCl, 10 HEPES, 0.1 EGTA, 4 Mg-ATP, 0.2 Na-GTP, 10 phosphocreatine (290–300 mOsm, pH 7.2–7.3). A liquid junction potential of −10 mV was not taken into account. Decay times of EPSPs were analysed by double exponential fit from average traces recorded during baseline and in PPDA. Only EPSPs not contaminated by low-threshold spikes or spontaneous events were considered. For this comparison, the membrane potential in PPDA was reset to baseline values by direct current (DC) injections. For Ca^2+^ current recordings in plasticity experiments, QX-314 was added to the intracellular solution, and CP-101.606 and PPDA were dissolved in DMSO that in the final solution had a concentration < 0.01%, to exclude unspecific inhibition of Ca_V_3 channels^78^. Ca^2+^ currents were elicited by 125-ms long hyperpolarizing steps from −50 mV to −100 mV. A baseline of stable Ca^2+^ currents of 5 min was measured in voltage-clamp before the cell was switched to current-clamp mode for conditioning, and voltage-clamp was resumed right after the end of the stimulation protocol. Current-clamp recordings were conducted with automatic bridge-balance of pipette resistance. Fluctuations in the membrane potential were corrected by DC injections. Layer 6 afferents were photostimulated with brief (0.1–1 ms) LED flashes (470 nm Optoled Lite, Cairn Research) controlled via a Master8 Pulse stimulator (A.M.P.Instruments). The intensity of the whole-field illumination was adjusted to elicit reliable monophasic currents (max 20 mW). Paired-pulse facilitation and constant response latency were monitored to exclude disynaptic activation of thalamic inputs onto nRt cells. The stimulation protocol for plasticity induction consisted of 10 stimuli at 10 Hz delivered every 30 s and repeated 30 times. Series resistance (R_s_) and input resistance (R_i_) were monitored throughout recordings by brief voltage and current pulses, for voltage- and current-clamp recordings, respectively, and data were rejected for R_s_ and R_i_ changes > 20%. Data were acquired through Digidata1320A/1322 A digitizers. Signals were amplified through a Multiclamp700B amplifier (Molecular Devices), sampled at 20 kHz and filtered at 10 kHz using Clampex10 (Molecular Devices). Clampfit10 (Molecular Devices) and Igor Pro 6 (WaveMetrics) were used for data analysis.

### *In vivo* electrophysiological recordings

During surgery, male mice of 6–10 weeks of age were anaesthetized with isoflurane. Two craniotomies were opened on the left hemisphere under stereotaxic coordinates to allow for subsequent acute positioning of the recording electrode in somatosensory cortex S1 and the optic fiber above the nRt (interaural coordinates, in mm: S1 +2.1 AP, +3.0 L; nRt +2.1 AP, +2.2 L). A head implant was fixed to the skull with cyanoacrylate glue and surrounded by a recording chamber in dental cement. A silver wire was fixed to the inner wall of the chamber to serve as a reference electrode during recording. After 1–2 days, mice were deeply anaesthetized with urethane (2-2.5 mg/g, i.p.) and fixed to the recording frame via the head implant. A tungsten microelectrode (FHC, 10–12 MΩ) was lowered into deep cortical layers of S1 (−0.98 mm, at 45°) for local field potential (LFP) recording, and an optic fiber (0.2 mm diameter) was slowly descended above the nRt (−2.8 mm) for optogenetic stimulation. The recording chamber was filled with 0.9% NaCl. For local drug injection in the thalamus, a 33 G Hamilton needle was lowered to the nRt and 0.5 µl of ACSF containing MK-801 and 25 mM Alexa 594 were slowly delivered at 3.2 mm and 3 mm from the cortical surface. At each injection site, MK-801 was injected at a dose of 4 µg. Subsequently, the needle was slowly retracted and the optic fiber was descended. The same procedure was used for control experiments with vehicle injections. Photoactivation of ChR2-expressing corticothalamic fibers was performed with 10 stimuli of 0.5 ms at 10 Hz delivered 30 times every 30 s with a 473 nm laser (Rapp Optoelectronics, ~10–50 mW at the exit of the optic fiber). At the end of each experiment, the recording site in S1 was marked by a brief electrocoagulation (50 µA, 8 s), and the animal was perfused intracardially with 4% paraformaldehyde dissolved in phosphate buffer after receiving an overdose of pentobarbital. Fluorescence imaging was performed on 100 µm-thick slices to verify the site of recording, the drug diffusion after local injection and the trajectory of the optic fiber in the tissue. Electrophysiological signals were acquired at 1 kHz with a Plexon MAP2 system (preamplifier board: 0.07–300 Hz; high impedance headstage: AC 0.8 Hz–54 kHz) and analysed with Igor 6 (Wavemetrics). Time-frequency decomposition was performed using a Morlet wavelet transform between 0.05 s and 2 s, corresponding to the frequency band 0.5–20 Hz, with a scale resolution factor of 0.125. Power spectra were averaged for periods of 20 min prior to and between min 10–30 after laser stimulation. Dominance of slow oscillations (SOs, 0.5–1.25 Hz) and delta oscillations (δ, 1.25–4 Hz) was defined for 4-s epochs in which the power in the respective frequency band was ≥ 50% of the total power. Limits of the frequency bands for SOs and δ oscillations were chosen based on the scale factor used for wavelet analysis, in order to balance the number of discrete frequency points used to calculate mean power values (12 points for SOs and 14 points for δ oscillations).

### Statistics

Statistical analyses were performed using Prism 6 (GraphPad Software) setting an alpha level of 0.05. The choice of parametric and non parameteric tests was based on normal distribution of the data (Shapiro-Wilk normality test). Comparison within series (drug action on synaptic currents and plasticity) was done with two-sided paired t test on absolute values. Comparison of LFP power spectra was performed on 1 Hz-binned data with two-way repeated measures ANOVA, for the factors frequency and stimulation, followed by *post hoc* Fisher’s LSD test. All data are presented as mean ± s.e.m.

### Data Availability

The datasets generated during and analysed during the current study are available from the corresponding authors on reasonable request.

## Electronic supplementary material

Supplementary Info
