# Supplementary material for: Cortical afferents onto the nucleus Reticularis thalami promote plasticity of low-threshold excitability through GluN2C-NMDARs
Source: Sci Rep. 2017 Sep 25;7:12271. doi: 10.1038/s41598-017-12552-8 (PMC5612942; doi:10.1038/s41598-017-12552-8)
Supplement: Supplementary file 1 — Supplementary Info [file 41598_2017_12552_MOESM1_ESM.pdf]

SUPPLEMENTARY INFORMATION

Cortical afferents onto the *nucleus Reticularis thalami* promote plasticity of low-threshold excitability through GluN2C-NMDARs

Laura M.J. Fernandez[1], Chiara Pellegrini[1], Gil Vantomme[1], Elidie Béard[1], Anita Lüthi[1,*] and Simone Astori[1,2,*]

[1] Department of Fundamental Neurosciences, University of Lausanne, 1005 Lausanne, Switzerland.

[2] Current address: Brain Mind Institute, École Polytechnique Fédérale de Lausanne, 1015 Lausanne, Switzerland.

* correspondence should be addressed to: simone.astori@epfl.ch or anita.luthi@unil.ch

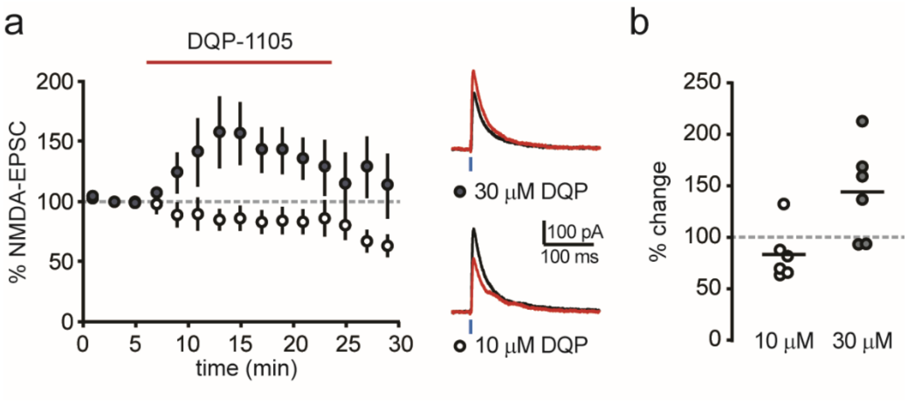

Figure S1 Effect of DQP-1105 on corticoreticular NMDA-EPSCs. (**a**) Change in peak amplitude of isolated corticoreticular NMDA-EPSCs upon superfusion of DQP-1105 (DQP) at 10 µM (white circles, n = 6) and 30 µM (black circles, n = 6), with representative traces shown on the right. (**b**) Summary of the effect of DQP at the tested concentrations, indicating inconsistent action of this compound on corticoreticular NMDA-EPSCs.

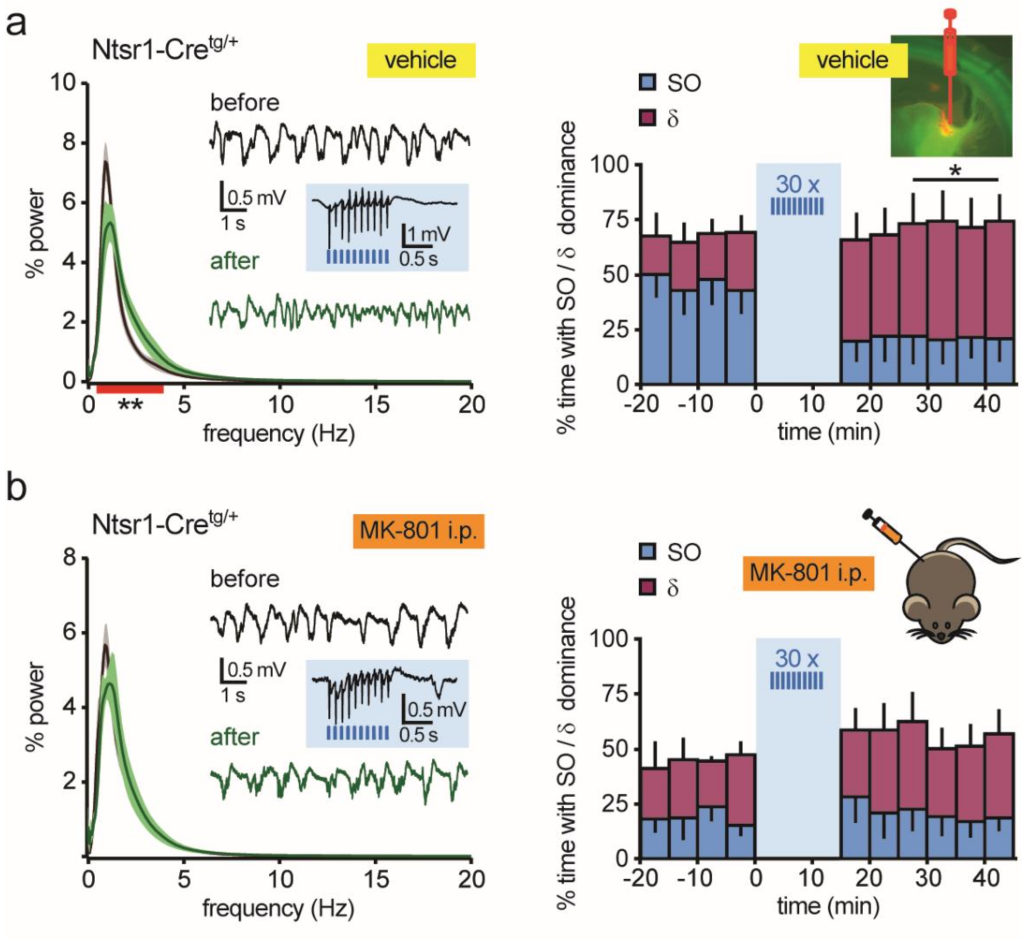

Figure S2 Modulation of thalamocortical oscillations *in vivo* with local thalamic vehicle injection and with systemic NMDAR-blockade. (**a**) Left, mean power spectra during baseline (black) at 10-30 min after train stimulation (green) in mice injected with vehicle (for MK-801) and Alexa 594 (n = 6). Red thick line indicates the frequency range where a significant difference occurred (two-way repeated measures ANOVA, $F_{17, 90} = 3.69$, $p < 0.0001$ for frequency x stimulation interaction, ** $p < 0.01$, *post hoc* Fisher's LSD test). Representative cortical LFP stretches and photoactivated responses are displayed in the insets. Right, time course of % dominance of SOs (blue) and δ oscillations (purple). * $p < 0.05$, Wilcoxon test. Fluorescence image shows verification of the injection site with Alexa 594 (red). (**b**) Same representation as in (**a**) for recordings with systemic injection of MK-801 (n = 5).
